# A Mobile Health Platform to Disseminate Validated Institutional Measurements During the COVID-19 Outbreak: Utilization-Focused Evaluation Study

**DOI:** 10.2196/18668

**Published:** 2020-04-14

**Authors:** Ido Zamberg, Sergio Manzano, Klara Posfay-Barbe, Olivier Windisch, Thomas Agoritsas, Eduardo Schiffer

**Affiliations:** 1 Division of General Internal Medicine Department of Medicine University Hospitals of Geneva Geneva Switzerland; 2 Faculty of Medicine University of Geneva Geneva Switzerland; 3 Division of Pediatric Emergency Department of Pediatrics University Hospitals of Geneva Geneva Switzerland; 4 Division of General Pediatrics Department of Pediatrics University Hospitals of Geneva Geneva Switzerland; 5 Division of Urology Department of Surgery University Hospitals of Geneva Geneva Switzerland; 6 Division of Anesthesiology Department of Anesthesiology, Clinical Pharmacology, Intensive Care and Emergency Medicine Geneva Switzerland

**Keywords:** covid-19, novel coronavirus, smartphone, SARS-COV-2, mHealth, knowledge, information, dissemination, health policy, infectious disease, outbreak, public health, preparation

## Abstract

**Background:**

As part of the response plans for the current outbreak of severe acute respiratory syndrome coronavirus 2 (SARS-CoV-2), authorities are drafting and implementing containment measures across jurisdictions worldwide in the effort to slow down transmission and reduce the infection rate. A solid communication strategy is needed to increase the reach of valid information to health professionals, reduce misinformation, and efficiently implement recommended measures.

**Objective:**

The aim of this paper is to describe the utilization of a dedicated mobile health (mHealth) platform to disseminate up-to-date and validated information about SARS-CoV-2 to all medical staff of the Children’s Hospital at the University Hospitals of Geneva.

**Methods:**

Three documents containing institutional information concerning screening, local containment procedures, and frequently asked questions and answers for parents were made available to the staff through a mobile app developed in the University of Geneva, Switzerland. Using a third-party statistics tool, we anonymously monitored user activity as well as content utilization patterns since the diagnosis of the first case of SARS-CoV-2 in Switzerland on February 25, 2020.

**Results:**

From February 25, 2020, to March 13, 2020 (18 days), information documents on SARS-CoV-2 were viewed 859 times, which accounted for 35.6% of the total content views (total views=332). User activity increased significantly with 50.8 (SD 14.4) users per day in this period as compared to the previous weeks (mean 26.4, SD 9.8; *P*<.001). In addition, session numbers per day more than doubled during the aforementioned period (*P*<.001). In a survey, medical staff found the information easy to find within the app. On a 10-point Likert scale, the ability of the app to reassure staff in clinical practice was rated as 7.6 (SD 2.1), time-saving ability was rated as 8.5 (SD 2.1), and the need to look for information from other sources was rated as 5.9 (SD 3.3).

**Conclusions:**

The use of an mHealth solution to disseminate novel coronavirus–related information seemed to be an effective and time-saving communication channel within our institution during the SARS-CoV-2 outbreak. Medical staff felt reassured and informed in daily practice. More research should be done on the clinical impact and outcomes of the integration of mHealth solutions as a communication channel of validated information within health institutions.

## Introduction

The global outbreak of the novel coronavirus severe acute respiratory syndrome coronavirus 2 (SARS-CoV-2) responsible for coronavirus disease (COVID-19) infection and its implications are still unfolding. With more than 800,000 confirmed cases worldwide [1], governments and local authorities are working on different response plans that are adapted to local epidemiology and resources in order to reduce the risk of community spread and slow down disease transmission [2].

Local and global response plans include classic outbreak measures such as travel restrictions to and from high-risk areas, social distancing, community containment, quarantine for confirmed or suspected cases, and cancellation of large-scale public events and gatherings [3-5]. On the personal level, the World Health Organization (WHO) is recommending hygiene measures such as avoiding contact with confirmed cases and hand washing, in an effort to reduce viral transmission rate [2].

In parallel, local hospitals and health authorities worldwide are bracing themselves for the possibility of a large influx of suspected and confirmed cases and aiming to prevent nosocomial transmission to patients’ families and health workers [2,3]. For example, in the Children’s Hospital at the University hospitals of Geneva, Switzerland, several local procedures were drafted to streamline patients sent for testing or medical care, diagnostic criteria were adopted and updated daily as per international consensus, and a contained screening facility was set up to separate possibly infected children from the rest of the hospital. In addition, a hotline was established and was rapidly overwhelmed.

In order to be efficient, disease containment measures require a solid communication strategy to avoid misinformation not only to the general public but also between global and local health authorities as well as within health institutions, so that validated guidance can properly reach local medical staff [6].

In fact, as seen in recent outbreaks of measles, Zika virus, and Ebola, the public is exposed to a large amount of information from both official channels such as the WHO and local authorities as well as from unofficial channels such as newspapers and social media [7-9], with obvious risk from the latter to provide confusion and misinformation [8]. Health professionals, who might have a more critical insight for these channels, would still find themselves exposed to a large amount of information for which validation could be lacking.

As mobile health (mHealth) solutions are becoming more relevant in health professions [10], the use of mobile devices via dedicated platforms as a means of communication may increase the reach of validated information to clinicians.

In this short paper, we describe our efforts to disseminate locally validated and up-to-date guidance about COVID-19 to the medical staff in the Children’s Hospital in the University Hospitals of Geneva through a mobile platform developed in the University of Geneva, Switzerland. Our hypothesis was that providing guidance through the mobile app would be perceived as time effective by medical staff and would provide reassurance in clinical practice, as validated information is readily available and updated regularly.

## Methods

Owing to our medical students’ and residents’ need to easily access locally endorsed and validated medical knowledge, we have developed a mobile platform called “HeadToToe” [11,12]. The platform provides an institutional knowledge dissemination solution and consists of iOS and Android mobile apps where medical students and health professionals can access medical content organized by medical specialties, such as local and international guidance, clinical skills videos, and administrative material. The platform provides daily practice and an administration interface accessed by delegated senior staff from each of the hospital’s departments, who select and validate content they deem important for continuous medical education. Content managers define revision dates and expiration dates for each item. Obsolete items are deleted automatically by the system. Automatic and anonymous statistics collection using Yahoo Flurry [13] provide data on user activity and content views patterns.

During the COVID-19 pandemic, the platform was used as a communication channel in the Children’s Hospital to disseminate local procedures, treatment plans, and general information about the novel coronavirus to health care workers, particularly physicians.

To assess the impact of such an mHealth information channel on clinical practice and provide feedback to medical leadership of its usefulness, we used a utilization-focused evaluation method [14]. We analyzed user activity and content use patterns collected by the platform since the introduction of outbreak measures in our institution. Data were collected on the average and total number of users and sessions per day, average usage time per user per day, and the total and specific number of content views per day.

In addition, we conducted an online survey among medical staff who used the platform during the same period. Survey questions focused on the impact of mHealth solutions on daily practice, specifically on time effectiveness and reassurance ability concerning a specific clinical challenge (care of patients with COVID-19).

*P* values were calculated using SAS JMP (SAS Institute Inc) with a *t* test for means. Values of *P*<.05 were considered significant.

## Results

**Figure 1:**
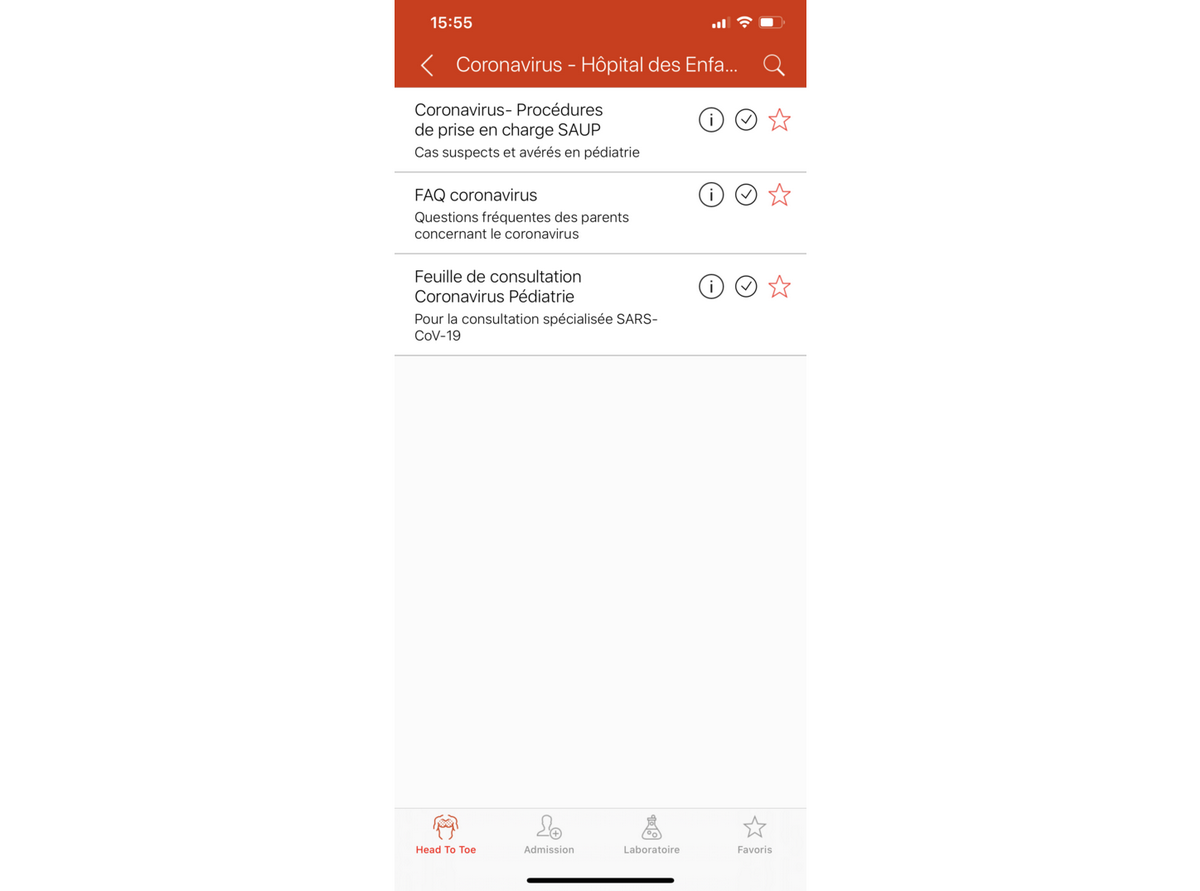
Mobile app user interface displaying the documents section with COVID-19 content.

Three novel coronavirus–related documents (Figure 1) were made available to the medical staff of the Children’s Hospital through the mobile platform: (1) institutional screening and containment procedures, (2) frequently asked questions and answers for parents, and (3) a standardized consultation sheet. The medical staff’s demographics are summarized in Table 1.

Since the first case of SARS-CoV-2 in Switzerland was announced on February 25, 2020, until March 13, 2020 (18 days), the mentioned documents were viewed 859 times. This amounted to 35.6% of total documents views from a total of 332 documents.

**Figure 2:**
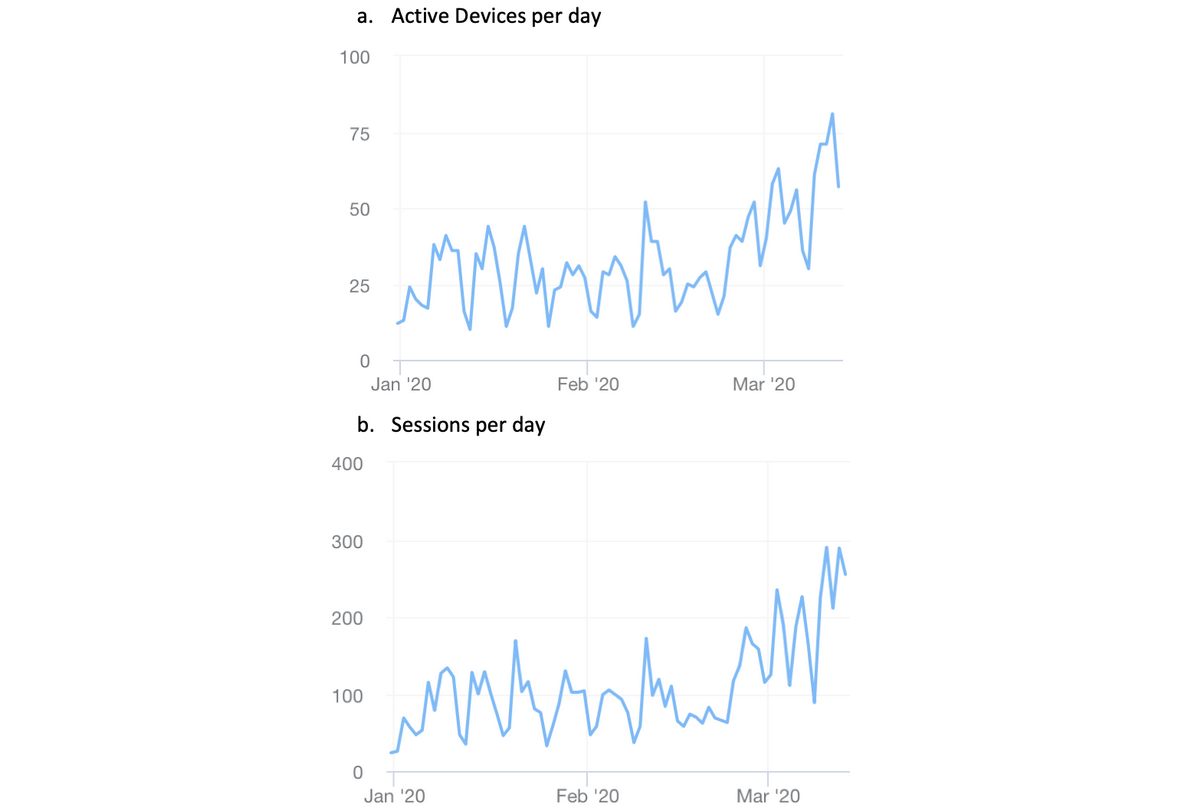
Mobile platform user activity between January 01, 2020, and March 13, 2020.

Concerning user activity (Table 2), we observed a significant increase (92%, *P*<.001) in the number of users per day (mean 50.8, SD 14.4) from January 1, 2020, to February 24, 2020, as compared to the previous weeks (mean 26.4, SD 9.8; Figure 2A). The number of sessions per day increased significantly (*P*<.001) and more than doubled in the aforementioned period with a mean of 182.9 (SD 60.0) sessions per day compared with 84.2 (SD 33.6) sessions per day in the previous weeks. (Figure 2B).

**Table 1:** Demographic characteristics of hospital staff (N=125).

Characteristic		Staff members, n (%)^a^
**Sex**		
	Male	34 (27.2)
	Female	87 (69.9)
**Age (years)**		
	25-30	28 (22.4)
	31-35	24 (19.2)
	36-40	18 (14.4)
	41-50	29 (23.2)
	51-60	24 (19.2)
	>60	2 (1.6)
**Profession**		
	Medical doctor	94 (75.2)
	Nurse	25 (20.0)
	Other^b^	6 (4.8)
**Medical unit affiliation**		
	Pediatric emergency room	41 (33.1)
	Intensive care and neonatology	14 (11.3)
	Ward	25 (20.2)
	Outpatient unit	30 (24.2)
	Other	14 (11.3)

^a^Total number may sometimes not add up to a 100%, as staff members were allowed to skip questions.

^b^Psychologists, caregivers, and administrators.

**Table 2:** User activity on the mobile platform during the SARS-CoV-2 outbreak.

Parameter		Value, mean (SD)		*P* value	
**Active users per day**					<.001
	Jan 1, 2020 to Feb 24, 2020		26.4 (9.8)		
	Feb 25, 2020 to Mar 13, 2020		50.8 (14.4)		
**Sessions per day**					<.001
	Jan 1, 2020 to Feb 24, 2020		84.2 (33.6)		
	Feb 25, 2020 to Mar 13, 2020		182.9 (60)		

In a survey answered by 125 health professionals of the Children’s Hospital, 93 staff members (75.0%) said that they are directly concerned with care of patients with SARS-CoV-2, and 84 (67.2%) said they downloaded the mobile app. Among staff who downloaded the platform, 70 (83.3%) said that information concerning SARS-CoV-2 was easy to find because of the app. On a 10-point Likert scale, the mHealth solution was rated 8.5 (SD 2.1) for being time saving and 7.6 (SD 2.1) for reassurance concerning care of patients with SARS-CoV-2 in daily practice. Finally, when asked for the need to seek other sources of information other than the mobile platform, medical staff rated the solution a score of 5.9 (SD 3.3; Table 3).

**Table 3:** Medical staff’s utilization of a dedicated mHealth solution for SARS-CoV-2 information seeking.

Question	Value, mean (SD)	Yes, n (%)	No, n (%)	Neutral , n (%)	Total, n
Do your clinical activity directly concern children with suspected or confirmed SARS-CoV-2 infection?	N/A^a^	93 (75.0)	31 (25.0)	1	124
Did you download the app “HeadToToe”?	N/A	84 (67.2)	41 (32.8)	-	125
Is information concerning SARS-CoV-2 easy to find thanks to “HeadToToe” app?^b^	N/A	70 (83.3)	-	14 (16.6)	84
Do you consider the utilization of this dedicated mHealth solution as timesaving for finding information concerning SARS-CoV-2?^b,c^	8.5 (2.1)	N/A	N/A	N/A	N/A
Did you feel the need to use other sources in order to find information concerning SARS-CoV-2?^b,c,d^	5.9 (3.3)	N/A	N/A	N/A	N/A
Did the use of dedicated mHealth solution for accessing information concerning SARS-CoV-2 reassured you in your clinical practice?^b,c^	7.6 (2.1)	N/A	N/A	N/A	N/A

^a^N/A: not applicable.

^b^Information presented for this question concern medical staff who downloaded the app.

^c^These questions are scored on a Likert scale from 0 to 10; 0 indicates the lowest score, 5 indicates a neutral score, and 10 indicates the highest score.

^d^0 - No need for other information sources, 10 - Important need for other information sources.

Among staff who felt the need to search for more information, 48 (42.5%) answered they used national government websites, 31 (27.4%) used dedicated websites of health institution (WHO, Centers for Disease Control and Prevention), 9 (8%) used nondedicated professional websites, and 19 (16.5%) used nonofficial sources such as newspapers and television; none declared using social media.

## Discussion

Communication strategies for sound clinical guidance are important for clinicians to choose evidence-based treatment plans, even more so in the times of infectious disease outbreaks, where misinformation can play a key role in failure of containment methods [6,8].

These strategies usually involve both information sources dedicated for the general public and sources targeted for health institutions and professionals, such as dedicated websites, scientific papers, and governmental procedures. Within an institution, local leadership often uses tools such as emails, posters, and conferences to reach and inform their staff [6]. These methods, however, have obvious flaws, especially in situations like the current one, of a newly discovered virus (SARS-CoV-2), where solid scientific evidence is still lacking and new, and sometimes contradictory, information is being published on a daily basis.

Moreover, in these situations, health professionals may have to deal with not only a growing amount of workload and patient consultations, but also the difficulty of critically appraising the vast amount of published information on the subject in order to make evidence-based decisions.

Therefore, it is crucial, in our opinion, that health institutions are able to not only communicate with local, national, and international authorities in order to create response plans and protocols during outbreaks, but also communicate these protocols to their medical staff in order to inform, reassure, and help them with clinical decision making. It is crucial as well that this information reaches as many clinicians as possible, with the possibility to keep them updated as new information unfolds.

Medical leadership’s dissemination of COVID-19 information through the mobile platform in our institution was answered with a significant increase in app usage and relevant content use. Medical staff found the information easy to find and the mHealth solution time saving with regard to COVID-19 information seeking. These results might provide more evidence for the time-saving benefits of mHealth solutions in daily practice.

Mobile information dissemination platforms, as used in our institution, may present an interesting communication method, especially in the era where smartphones are ubiquitous among clinicians [10]. User activity and content monitoring in real time may provide institutional leadership with valuable information regarding staff’s information needs as well as information dissemination efficiency. In our institution, increased user activity and content views in the Children’s hospital motivated medical leadership to produce and disseminate more COVID-19–related material through the mobile platform. In addition, due to abovementioned results, institutional leadership decided to deploy the mobile platform within all medical departments in order to disseminate institutional COVID-19 content to all of the hospital’s medical staff.

mHealth solutions such as the one presented here may help in solving some of the presented challenges by increasing the reach of information for health professionals and thus decreasing misinformation and confusion, as key information is centralized in one platform and validated, up-to-date information is easy to find. Moreover, due to the administration interface, leadership was easily able to update information, and users have access only to the latest version of relevant content. These milestones would be harder to achieve with classic methods as emails, which may be hard to sort and find, or with printed material, especially when frequent content updating is necessary.

Our study’s main limitation is our inability, at this stage, to provide evidence of the impact of this mHealth intervention on the quality and outcomes of patient care.

In conclusion, while more data are needed to study the short- and long-term clinical impact and outcomes of this type of mHealth intervention, the use of a mobile platform designed to disseminate information during the SARS-CoV-2 outbreak seems to be an effective and time-saving method for communicating local guidance within our institution. Medical staff felt reassured and informed about procedures for care of patients with SARS-CoV-2 and seemed to have less need to seek other sources of information.

## Abbreviations

COVID-19: coronavirus disease
mHealth: mobile health
SARS-CoV-2: severe acute respiratory syndrome coronavirus 2
WHO: World Health Organization

## References

[1] (2020). World Health Organization.

[2] Sohrabi C, Alsafi Z, O'Neill N, Khan M, Kerwan A, Al-Jabir A, Iosifidis C, Agha R (2020). World Health Organization declares global emergency: A review of the 2019 novel coronavirus (COVID-19). Int J Surg.

[3] Fisher D, Heymann D (2020). Q&A: The novel coronavirus outbreak causing COVID-19. BMC Med.

[4] (2020). World Health Organization.

[5] Wu Z, McGoogan JM (2020). Characteristics of and Important Lessons From the Coronavirus Disease 2019 (COVID-19) Outbreak in China: Summary of a Report of 72 314 Cases From the Chinese Center for Disease Control and Prevention. JAMA.

[6] Dearinger AT, Howard A, Ingram R, Wilding S, Scutchfield D, Pearce KA, Hall B (2011). Communication efforts among local health departments and health care professionals during the 2009 H1N1 outbreak. J Public Health Manag Pract.

[7] Chandrasekaran N, Gressick K, Singh V, Kwal J, Cap N, Koru-Sengul T, Curry CL (2017). The Utility of Social Media in Providing Information on Zika Virus. Cureus.

[8] Gesser-Edelsburg A, Diamant A, Hijazi R, Mesch GS (2018). Correcting misinformation by health organizations during measles outbreaks: A controlled experiment. PLoS One.

[9] Mwesiga A (2011). Reporting epidemics: newspapers, information dissemination and the story of Ebola in the Ugandan district of Luweero. Pan Afr Med J.

[10] Gagnon M, Ngangue P, Payne-Gagnon J, Desmartis M (2016). m-Health adoption by healthcare professionals: a systematic review. J Am Med Inform Assoc.

[11] Windisch O, Zamberg I, Iselin C, Schiffer E (2019). Head To Toe, a medical knowledge distribution platform : a practical example in urology. Article in French. Rev Med Suisse.

[12] Zamberg I, Windisch O, Agoritsas T, Nendaz M, Savoldelli G, Schiffer E (2020). HeadToToe: A Mobile Medical Knowledge dissemination platform: strengths, limitations and preliminary usage assessment. JMIR Med Educ.

[13] Flurry.

[14] Patton MQ (2000). Utilization-Focused Evaluation. Evaluation Models. Evaluation in Education and Human Services.

